# Cardiac and Vascular Sympathetic Baroreflex Control during Orthostatic Pre-Syncope

**DOI:** 10.3390/jcm8091434

**Published:** 2019-09-10

**Authors:** Raffaello Furlan, Karsten Heusser, Maura Minonzio, Dana Shiffer, Beatrice Cairo, Jens Tank, Jens Jordan, André Diedrich, Peter Gauger, Antonio Roberto Zamuner, Franca Dipaola, Alberto Porta, Franca Barbic

**Affiliations:** 1 Department of Internal Medicine, Humanitas Clinical and Research Center, IRCCS, Humanitas University, 20089 Rozzano, Italy; maura.minonzio@humanitas.it (M.M.); dana.shiffer@humanitas.it (D.S.); franca.barbic@humanitas.it (F.B.); 2 Institute of Aerospace Medicine, German Aerospace Center (DLR), 51147 Cologne, Germany; Karsten.Heusser@dlr.de (K.H.); Jens.Tank@dlr.de (J.T.); Jens.Jordan@dlr.de (J.J.); Peter.Gauger@dlr.de (P.G.); 3 Department of Biomedical Sciences for Health, University of Milan, 20122 Milan, Italy; beatrice.cairo@unimi.it (B.C.); alberto.porta@unimi.it (A.P.); 4 Chair of Aerospace Medicine, University of Cologne, 51147 Cologne, Germany; 5 Autonomic Dysfunction Center, Vanderbilt University Medical Center, Nashville, TN 37232, USA; andre.diedrich@vumc.org; 6 Departamento de Kinesiología, Universidad Católica del Maule, 3530000 Talca, Maule, Chile; beto.zam@gmail.com; 7 Department of Cardiothoracic, Vascular Anesthesia and Intensive Care, IRCCS Policlinico di San Donato, 20097 San Donato Milanese, Italy

**Keywords:** syncope, vasovagal, baroreceptors, muscle sympathetic nerve activity, power spectrum analysis, heart rate variability, blood pressure variability, MSNA variability

## Abstract

We hypothesized that sympathetic baroreflex mediated uncoupling between neural sympathetic discharge pattern and arterial pressure (AP) fluctuations at 0.1 Hz during baroreceptor unloading might promote orthostatic pre-syncope. Ten volunteers (32 ± 6 years) underwent electrocardiogram, beat-to-beat AP, respiratory activity and muscle sympathetic nerve activity (MSNA) recordings while supine (REST) and during 80° head-up tilt (HUT) followed by −10 mmHg stepwise increase of lower body negative pressure until pre-syncope. Cardiac and sympathetic baroreflex sensitivity were quantified. Spectrum analysis of systolic and diastolic AP (SAP and DAP) and calibrated MSNA (cMSNA) variability assessed the low frequency fluctuations (LF, ~0.1 Hz) of SAP, DAP and cMSNA variability. The squared coherence function (K^2^) quantified the coupling between cMSNA and DAP in the LF band. Analyses were performed while supine, during asymptomatic HUT (T_1_) and at pre-syncope onset (T_2_). During T_2_ we found that: (1) sympathetic baroreceptor modulation was virtually abolished compared to T_1_; (2) a progressive decrease in AP was accompanied by a persistent but chaotic sympathetic firing; (3) coupling between cMSNA and AP series at 0.1 Hz was reduced compared to T_1_. A negligible sympathetic baroreceptor modulation during pre-syncope might disrupt sympathetic discharge pattern impairing the capability of vessels to constrict and promote pre-syncope.

## 1. Introduction

According to the recent European Society of Cardiology guidelines, syncope is a transient loss of consciousness, due to a temporary cerebral hypoperfusion, characterized by rapid onset, short duration and spontaneous complete recovery [1]. Vasovagal syncope triggered by gravitational stimuli, i.e., in the upright position, is common accounting for up to 74% of total syncope seen in emergency departments [2]. The exact mechanisms underlying orthostatic vasovagal syncope are still elusive [3,4]. Baroreflex dysfunction appears to be crucial [3] in promoting hypotension and/or bradycardia through neural sympathetic withdrawal and/or cardiac vagal over-activity. However, the relationships among transient baroreflex abnormalities, autonomic modifications and target organs responses, i.e. heart rate and arterial pressure changes, are yet to be elucidated.

In healthy individuals, the assumption of the up-right position is associated with blood pooling in the lower body capacitance vessels which rapidly reduces venous return, cardiac output, and arterial pressure [5,6]. These hemodynamic changes produce cardiopulmonary and arterial baroreceptor unloading, in turn decreasing afferent baroreceptor traffic to the brain stem. In healthy individuals, the subsequent reflex increase in cardiovascular sympathetic activity [3] results in an enhancement of the neural sympathetic firing to resistance vessels (muscle sympathetic nerve activity, MSNA), of plasma norepinephrine and heart rate (HR) with maintained blood pressure values [7].

Remarkably, healthy cardiovascular regulation during upright posture is characterized by synchronized sympathetic discharge pattern to the vessels and spontaneous arterial pressure and heart rate fluctuations with a period of about 10 s, i.e., at 0.1 Hz [8]. Indeed, in healthy volunteers rhythmic carotid baroreceptor perturbations at 0.1 Hz by a neck suction device resulted in corresponding changes in blood pressure and sympathetic efferent nerve firing [9]. Therefore, in healthy humans maintaining the upright position, sympathetic vascular discharge activity, heart rate, and arterial pressure seem to share common oscillatory patterns characterized by high levels of inter-signal coupling at 0.1 Hz, as assessed by the coherence function [8,10] or phase synchronization index [11]. 

In contrast, patients with afferent baroreflex failure [12,13,14] exhibited either orthostatic hypertension [12,15] or orthostatic hypotension [13]. A woman suffering from baroreflex failure secondary to neck irradiation for cancer management experienced remarkable orthostatic hypotension in the face of substantially raised plasma norepinephrine levels and MSNA compared to the supine position [13]. In this patient, the sympathetic firing activity was chaotic suggesting that such a disorganized vascular sympathetic activation may tonically raise circulating norepinephrine but nevertheless fails to maintain arterial pressure in the upright position [13]. Transient reduction or loss of cardiac [3,5,16] and sympathetic baroreflex control [5,17,18,19] and of cardiopulmonary baroreceptor modulation of sympathetic firing [5] have been observed before orthostatic vasovagal syncope. 

Altered baroreflex control before vasovagal syncope should attenuate the 0.1 Hz coupling between heart rate, arterial pressure, and MSNA variability [18,20,21]. The mechanism could promote hypotension since arterial vasoconstriction to neural vasomotor stimulation is limited to a narrow frequency range with an optimum around 0.1 Hz in humans [22,23]. 

Therefore, we tested the hypothesis that transiently impaired cardiac and vascular sympathetic baroreflex control before pre-syncope may be associated with uncoupling between neural sympathetic discharge activity and arterial blood pressure fluctuations at ~0.1 Hz. Moreover, we hypothesized that these neural and hemodynamic changes may promote hypotension and pre-syncope.

## 2. Material and Methods

### 2.1. Experimental Protocol

The present study has been realized as a part of the European Space Agency Medium-Term-Bedrest Study (ClinicalTrials.gov Identifier: NCT01655979) and was conducted at the Institute of Aerospace Medicine of the German Aerospace Center (DLR) [24] in Cologne. Ten healthy men (32 ± 6 years, BMI 23.4 ± 1.6 kg/m^2^) participated in the study. All the volunteers gave their written informed consent before the study commenced. The protocol adhered to the principles of the Declaration of Helsinki and was approved by the ethics committee of the Aerztekammer Nordrhein (Approval #2008294, Dusseldorf, Germany).

Electrocardiogram (ECG), noninvasive beat-by-beat arterial pressure (AP) (Finapres Medical Systems, The Netherlands), respiration activity (Electrobioimpedance Amplifier, Biopac Systems Inc., Santa Barbara, CA, USA), and MSNA (Nerve Traffic Analyzer model 662C-3, University of Iowa Bioengineering, Iowa City, IA, USA) were continuously recorded with BNC-2110 data acquisition system and LabVIEW 7.0 software (National Instruments, Austin, TX, USA). MSNA was recorded from the peroneal nerve of the right leg as detailed elsewhere [25]. Briefly, multiunit recordings of postganglionic sympathetic activity were obtained by placing a tungsten electrode in a fascicle of the right peroneal nerve, posterior to the fibular head. A reference electrode was inserted subcutaneously, close to the recording needle. The raw neural signal was amplified (1000 × 99.9), fed to a band pass filter (bandwidth between 700 and 2000 Hz), rectified and integrated (time constant, 0.1 s) by a nerve traffic analyzer system (Bioengineering Department, University of Iowa, Iowa City, IA, USA). In one subject, the microneurography signal was lost when the 80° head-up tilt had been reached, and another subject had an insufficient signal to noise ratio recording, i.e., lower than 3, making the automatic analysis of MSNA impossible. Thus, data related to MSNA recordings are referred to eight individuals.

Data were recorded in all volunteers while supine (REST) and during fifteen minutes of 80° head-up tilt (HUT). Given that none exhibited pre-syncope at the end of HUT, additional three minutes of −10 mmHg stepwise increasing lower body negative pressure (LBNP) was applied until symptoms and signs of pre-syncope were evoked. 

Pre-syncopal symptoms included pallor, lightheadedness, blurred vision, sweating, or nausea. Pre-syncopal signs were defined as a decrease of HR > 80% and/or a decrease of systolic arterial pressure (SAP) > 40% compared to HUT. In the presence of one of the pre-syncope symptoms and signs the testing was interrupted by the physician in charge.

### 2.2. Data Analysis

ECG, arterial pressure (AP), respiratory activity, and MSNA were digitized at 500 Hz by an analog-to-digital converter (AT-MIO 16E2; National Instruments, Austin, TX, USA) and recorded on the hard disk of a personal computer for off-line analysis. Systolic arterial pressure (SAP) was computed as the maximum AP in a given heart period approximated as the temporal distance between two successive R-wave peaks detected in the ECG. Diastolic arterial pressure (DAP) was computed as the minimum arterial pressure following SAP. The temporal occurrences of the MSNA burst and DAP were also stored. Data analysis was performed on 5 minutes of supine position (REST), on 5 min of HUT in the absence of orthostatic intolerance symptoms (T_1_) and on the period just preceding the interruption of HUT (T_2_) because of the onset of pre-syncope signs and/or symptoms, as described in the experimental protocol. The time series length of REST and T_1_ comprised 300 consecutive beats. The duration of T_2_ was kept shorter than that of REST and T_1_ to operate in the “quasi” stationary conditions, imposed by the stepwise protocol and by the duration of the pre-syncope symptoms and signs, the onset of which forced the break of the gravitational stimulus. The mean duration of T_2_ was 2 min. 

### 2.3. Power Spectral Analysis of RR Interval and SAP Variability

Indices of cardiovascular autonomic profile were obtained by RR interval, SAP and respiratory activity variabilities, as assessed by power spectrum analysis techniques, described in detail elsewhere [26,27]. Two major oscillatory components can be identified and quantified from RR interval variability. The high frequency (HF_RR_) component (0.15 Hz to 0.4 Hz), a recognized index of the vagal efferent modulation directed to the sinoatrial node [27], and the low frequency (LF_RR_) component (0.04 Hz to 0.15 Hz), which reflects sympathetic and vagal modulation. During orthostatic stress normalized LF_RR_ is thought to primarily reflect the sympathetic modulation of the sinoatrial node activity and of its changes [8,26], although its functional meaning is still debated [28,29]. The LF_RR_/HF_RR_ ratio, a dimensionless index, assesses the sympatho-vagal relationship modulating the cardiac sinoatrial node [8,26]. The low frequency oscillatory component of SAP variability, LF_SAP,_ is an indirect index of the sympathetic vasomotor control [8,30]. 

### 2.4. MSNA Assessment and Calibrated MSNA (cMSNA) Series

Sympathetic bursts were detected by an adaptive threshold method where the burst detection threshold was updated on a beat-to-beat basis to follow baseline wandering and physiological variations of the MSNA burst amplitude [9]. The MSNA peaks overcoming the adaptive threshold were considered as MSNA bursts. To account for the latency from the AP sensing to the possible sympathetic response [31,32], the MSNA burst was searched in a temporal window ranging from 0.9 to 1.7 s starting from the first R-wave peak delimiting the current cardiac cycle [9]. 

Tonic MSNA markers were evaluated in the time domain and expressed in bursts/minute or bursts/100 beats.

In the present study the calibrated MSNA (cMSNA) was used to assess the MSNA variability and its link with the cardiovascular rhythms. As reported in detail by Marchi et al. [33], the cMSNA values were obtained as the number of detected MSNA bursts in a running window of 5 s divided by the window length. The resulting time series preserves dimensionality typical of an integrated neural discharge [33] (i.e., bursts per second). The beat-to-beat variability of cMSNA series allows the assessment of the modulation of the burst rate as operated by the sympathetic control. As a consequence of the definition of the cMSNA series, the mean of cMSNA is expressed as bursts/s. 

Phasic MSNA markers were assessed in the frequency domain analysis from the cMSNA variability.

In the present study, phasic cMSNA indexes were computed in the low frequency (LF, 0.04–0.15 Hz) and in the high frequency (HF, ~0.25 Hz) band.

### 2.5. Relationship between MSNA and DAP Oscillations in the Frequency Domain

Autoregressive spectrum analysis of DAP and cMSNA variability provided the power of the LF and HF fluctuations of DAP (LF_DAP_ and HF_DAP_) and cMSNA variability (LF_cMSNA_ and HF_cMSNA_) [8,33,34]. The squared coherence function (K^2^) quantified the amount of linear coupling between oscillatory components centered at the same frequency in different signal variabilities. In particular, cross-spectral analysis allowed the calculation of K^2^_cMSNA-DAP_, computed as the ratio of the cMSNA-DAP square cross-spectrum modulus divided by the product of the power spectra of cMSNA and DAP series [8,35]. K^2^_cMSNA-DAP_ was sampled in correspondence with the central frequency of the DAP components detected in the LF and HF band, K^2^_cMSNA-DAP_(LF) and K^2^_cMSNA-DAP_(HF) [35]. Cross-spectrum and power spectra involved in the calculation of K^2^_cMSNA-DAP_ were assessed by a bivariate autoregressive approach. The coefficients of the bivariate autoregressive model were identified via traditional least squares method and the model order was fixed to 10 [35].

The stationarity of the selected sequence was tested according to Magagnin et al. [36] over the original series after linear detrending. If the test for the steadiness of mean and variance was not fulfilled, a new selection was carried out again until the fulfillment of the prerequisites for restricted weak stationarity [36]. Testing the stationarity of the mean is necessary even after linear de-trending. Indeed, the cardiovascular variability trends are more complex and are not fully addressed by a simple linear approach.

### 2.6. Cardiac Baroreflex Modulation (cBRS)

The cardiac baroreflex sensitivity (cBRS) was assessed by two approaches:

In the frequency domain by the alpha index (α) [8,26] as a square root of the ratio between LF_RR_ and LF_SAP_. 

In the time domain according to the baroreflex sequence analysis [35,37,38]. The sequence analysis searches for sequences characterized by the contemporaneous increase (positive sequence) or decrease (negative sequence) of RR and SAP values. The slope of the regression line between each pairing of SAP and RR values was calculated over each sequence. cBRSseq corresponds to the average of all considered slopes and is expressed as ms/mmHg. 

### 2.7. Sympathetic Neural Discharge Baroreflex Modulation (sBRS)

Assessment of sBRS was based on how the DAP value relates to the occurrence of an MSNA burst, accounting for the baroreflex latency [39]. sBRS gain was assessed by the slope of the regression line between DAP values grouped into bins of 1 mmHg and the percentage of times we detected a MSNA burst associated to the considered values of DAP. Notably, the correlation coefficient (r_sBRS_) had to be significant, i.e., *p* < 0.05 [18,40]. Since the slope is a negative value, the steeper the slope the higher the sBRS gain. Conversely, when the slope tends to 0 (flatter pattern), then the sBRS gain decreases. Therefore, a flattening of the DAP-MSNA relationship implies a loss of baroreceptor-mediated sympathetic vasomotor control.

### 2.8. Statistics

Continuous variables are expressed as mean ± standard deviation. The normality of data was tested via the Shapiro-Wilk test. One-way ANOVA for repeated measures followed by Holm–Sidak’s post hoc test was used. The level of significance was set at 5%. SigmaPlot 11 (Systat Software Inc., Chicago, IL, USA) was used to perform the statistical analysis. 

## 3. Results

**Figure 1:**
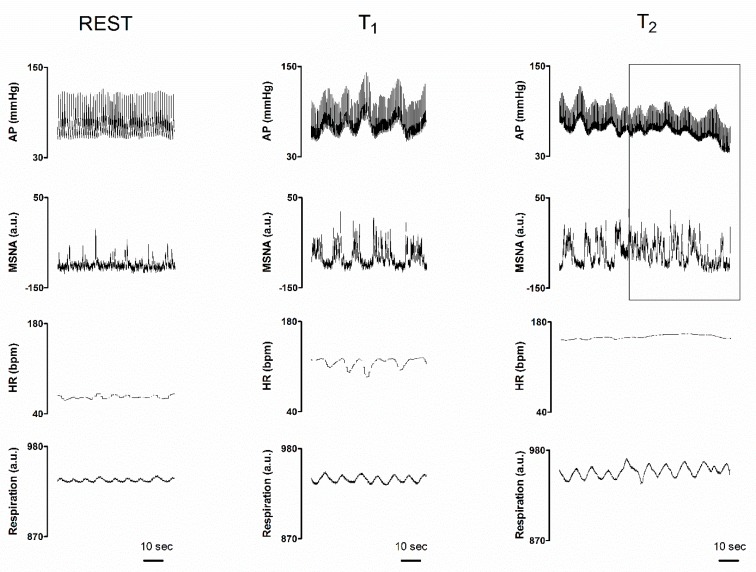
Individual traces of arterial pressure (AP), integrated muscle sympathetic nerve activity (MSNA), heart rate (HR) and respiratory activity (Respiration), recorded in a representative subject while supine (REST), during the asymptomatic phase of a 80° head-up tilt (T_1_) and during late tilt just before the pre-syncope onset (T_2_). The right squared window highlights the pre-syncope changes: notice the enduring but chaotic neural sympathetic discharge activity as compared with MSNA trace at T_1_ characterized by rhythmic sympathetic firing at 0.1 Hz (i.e., a cycle every 10 s); also, please note the concomitant progressive hypotension in spite of the compensatory tachycardia and sympathetic neural activity to the vessels (see above for further explanations).

Figure 1 illustrates spontaneous rhythmic oscillations of sympathetic neural discharge, respiratory activity and hemodynamic parameters recorded in a representative volunteer at rest and during the asymptomatic (central panels, T_1_) and pre-syncope phase (right panels, T_2_) of the 80° upright tilt and LBNP additional stimulus. During asymptomatic tilt, the sympathetic discharge activity (MSNA) was grouped in series of bursts occurring about every 10 s, i.e., at 0.1 Hz. This discharge pattern was linked to previous AP valleys. Similarly, HR increased with each blood pressure fall and decreased with blood pressure increase. When the subject experienced pre-syncopal symptoms and signs including nausea, pallor and sweating (symptomatic tilt, T_2_), sympathetic discharge activity while still being present became disorganized compared to T_1_, as highlighted by the drawn squared window. In spite of such a persistent sympathetic discharge activity to the vessels, arterial pressure decreased and tended to lose its rhythmic 10 s spontaneous fluctuations. Instead, arterial pressure fluctuations coupled to respiration became evident (squared window on the right panels). During T_2,_ HR slightly increased compared to asymptomatic tilt.

Table 1 summarizes the mean values of the hemodynamic parameters, respiratory activity and neurophysiological estimates of sympathetic activity at rest, during the asymptomatic (T_1_) and symptomatic (T_2_) phases of tilt, in the studied population. During T_1_, MSNA in burst/min remarkably increased, systolic and diastolic arterial pressures and respiratory rate remained stable, whereas RR interval was reduced. During T_2_, neural sympathetic discharge activity to the vessels was still high, whereas blood pressure significantly decreased along with R-R interval, i.e. the temporal distance between two consecutive R-wave peaks, although not significantly, compared to T_1_. Respiratory rate was similar in the three conditions.

Table 2 summarizes changes in cardiac baroreceptor gain (α index and cBRSseq) and the index of sympathetic baroreceptor modulation (sBRS) observed in the population at rest, during T_1_ and T_2_. Notice that both indices declined during T_1_ and further decreased during T_2_. In addition, the decrease was significant for the index of sympathetic baroreflex modulation (sBRS) which tended to zero. This suggests a virtual absence of baroreceptor inhibitory influence on the vascular sympathetic control in the pre-syncope phase.

Results of the frequency domain analysis of RR interval, SAP and cMSNA variability are synthesized in Table 3. Compared with the supine position, the gravitational challenge (T_1_) increased the LF/HF_RR_ ratio suggesting a prevailing cardiac sympathetic modulation, enhanced LF_SAP_ pointing to an increased sympathetic vasomotor control and decreased HF_RR_ suggesting a decline of the cardiac vagal modulatory activity, as expected. During asymptomatic T_1_, the power of the LF oscillatory component of cMSNA variability (LF_cMSNA_) increased, whereas HF_cMSNA_ slightly decreased compared to rest. The linear relationship between the spontaneous fluctuations in the low frequency band of cMSNA and DAP, as assessed by K^2^_MSNA-DAP_(LF), was greater than 0.5 during T_1_, suggesting an enhanced coupling between the two signals compared to rest. Compared to T_1_, during T_2_ there was a slight reduction in the LH/HF_RR_ ratio and in the power of the low frequency components of RR interval (LF_RR_ in n.u.), SAP and cMSNA variability with a concomitant tendency to shift the remaining power towards the HF oscillatory components. Accordingly, the linear coupling between LF_DAP_ and LF_cMSNA_, i.e., K^2^ cMSNA-DAP (LF), significantly decreased compared to T_1_. 

**Figure 2:**
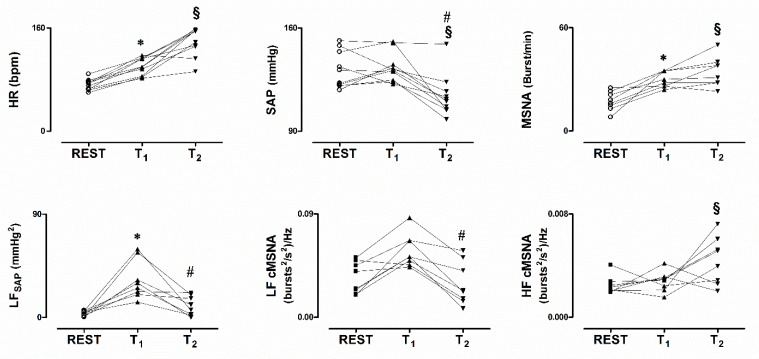
Individual values of heat rate (HR), systolic arterial pressure (SAP) and MSNA while supine (REST), during asymptomatic 80° head-up tilt (T_1_) and during pre-syncope (T_2_), in the upper panels. In the lower panels values of the low frequency spontaneous oscillation of SAP (LF_SAP_) and MSNA variability (LF_cMSNA_) are depicted. Notice hypotension in the presence of persistently elevated HR and MSNA during T_2_. In addition, T_2_ was characterized by a decrease of the power of the low frequency components of both SAP and cMSNA variability. * *p* < 0.05 REST vs T_1_. # *p* < 0.05 T_1_ vs T_2_. § *p* < 0.05 REST vs T_2_.

In the upper panels of Figure 2, the individual changes in HR, SAP and MSNA are depicted, as observed in the study population during the three intervals of interest. During T_2_ notice that both MSNA and HR were maintained in the presence of the SAP decline, compared to T_1_. These changes were consistent in almost all subjects. As depicted in the upper middle panel, notice that the subject with the greatest SAP values did not show a significant decline of SAP during T_2_. This subject complained from an early onset of orthostatic intolerance symptoms. Thus, the gravitational stimulus was broken off by the physician in charge before the development of significant hypotension. 

## 4. Discussion

The main finding of the present study is that during pre-syncope, cardiac baroreflex gain decreased further and sympathetic baroreceptor modulation was virtually abolished compared to asymptomatic HUT and the supine position. These changes were associated with a further mild decrease in RR interval and progressive significant blood pressure reduction despite persistently high but chaotic sympathetic discharge activity to the vessels (MSNA). These neural and hemodynamic modifications were associated with a loss of the linear coupling at 0.1 Hz between the sympathetic firing pattern (LF_cMSNA_) and vascular oscillatory responses indicated by LF_DAP_ and LF_SAP_.

We took advantage of the strict protocol that was set to address the problem of pre-syncope within a much larger study aimed at assessing the medium-term effects of bed confinement, granted by the European Space Agency [24]. Indeed, our healthy volunteers underwent a HUT maneuver up to 80°, followed by a progressive −10 mmHg lower body negative pressure administration until the onset of pre-syncope signs and symptoms. Therefore, we could reproduce a pre-syncope in all volunteers, independently of their individual orthostatic tolerance [1,41,42]. 

The results of the current study are in keeping with previous observations suggesting that the gains of both cardiac [3,5,16] and sympathetic baroreceptor control [5,16,17,20] decrease before syncope. However, in previous investigations arterial pressure modifications were obtained by infusing vasoactive drugs such as phenylephrine or nitroprusside. These compounds per se might have influenced carotid arteries wall tension, thus affecting the final response of carotid and aortic stretch receptors. In contrast, we assessed the cardiac baroreceptor sensitivity both in the time domain, by evaluating the spontaneous changes in RR interval as a response to arterial pressure modifications, and in the frequency domain by the index alpha, as previously used in vasovagal syncope by others [43]. The index alpha takes into account spontaneous fluctuations of heart rate and arterial pressure at 0.1 Hz, being therefore independent of external interventions. Similarly, we used the probabilistic approach [39,40] based on the likelihood of sympathetic burst occurrence per mmHg of diastolic arterial pressure spontaneous changes to assess sympathetic baroreflex control. In a preliminary study [18], the methodology proved to be effective in tracking baroreceptor sympathetic modulation before syncope. In keeping with our data, the gain of carotid-cardiac and carotid-vascular baroreflex response curves were found to be impaired before syncope in individuals undergoing a pressure perturbation of the carotid areas of the neck during a tilt test maneuver [44].

A novel technical feature of the present study is that the series derived from MSNA variability are intrinsically calibrated [33,40]. This was obtained on a beat-to-beat basis by means of a time moving window of 5 s and the assessment of the number of bursts within such a window by an adaptive threshold procedure, thus preserving the dimensionality of the integrated neural discharge activity, i.e., bursts per unit of time [33]. Such a method may overcome the limits of a more traditional approach based on the use of a simple low-pass filtering [8,34]. The latter may potentially alter the burst area and amplitude. In addition, this strategy makes the MSNA series sensitive to factors other than the neural traffic such as the position of the electrode, its proximity to the bundle, number of active units, amplification gain, and effects of noise superposed to the MSNA signal. In order to limit the effect of those confounders, MSNA series require the normalization of any index to that derived from a baseline condition and to assess the markers as percent variation. However, any normalization introduces the dependency of the result on the selection of the control condition and the normalization algebra, the most utilized metric being the ratio of the marker computed in a given condition to that in the control session [9]. The application of this strategy enabled us to find an association between the low frequency (LF) power of SAP variability and the normalized LF power of the MSNA series [34] and its increase during an orthostatic challenge [8]. Conversely, the present calibrated beat-to-beat variability series of MSNA is expressed in typical units of the integrated neural activity signal (i.e., burst per seconds) and represents the modulation of the frequency of the MSNA bursts. Remarkably, the LF power of this calibrated MSNA series was correlated with tilt table angles during HUT and to the LF power of SAP [33].

A striking finding of the present study was the progressive decline in blood pressure during pre-syncope in the presence of tachycardia and a persistent neural sympathetic firing to the vessels. The coupling between diastolic arterial pressure and MSNA before syncope was previously addressed in young adults by the use of the phase synchronization index [11]. This index increased during early tilt compared to supine position but dramatically declined before orthostatic syncope, pointing to a loss of synchronization between the two variables, in keeping with our observation. In the current study, MSNA was even slightly greater during pre-syncope than during the asymptomatic phase of tilt.

Taken together these observations deserve some comments. 

First, while in the past, vasovagal syncope was described as being preceded by sympathetic neural silence [5,16,45,46], more recently other authors observed persistent MSNA before the loss of consciousness [20,46,47,48,49]. These findings suggest that mechanisms other than complete neural sympathetic withdrawal may promote orthostatic vasovagal syncope. However, in these studies it is unclear whether a complete loss of consciousness was achieved in the patients. 

As to the current investigation, all subjects reached the pre-syncope stage with the combination of the tilt maneuver and the LBNP procedure, with orthostatic intolerance symptoms, significant hypotension and sometimes bradycardia were present and prompted the break of the orthostatic challenge. None of the subjects lost consciousness. Indeed, according to European Space Agency (ESA) policy, in the present study the gravitational stimulus was interrupted by the physician in charge who acted as a third party, not being part of the study team.

Second, while the neural sympathetic firing pattern was organized after a prevailing discharge activity at 0.1 Hz during the asymptomatic phase of tilt, thus mimicking analogous spontaneous fluctuations in heart rate [8] and systolic arterial pressure [8,10], the pre-syncope period was characterized by a highly disordered sympathetic activity to the vessels. This phenomenon is evident by visual inspection in the current and in previous studies by Cooke et al. [20] and Kamiya et al. [10]. Moreover, in the present study we observed a decrease of the LF_cMSNA_ oscillatory component of MSNA variability during pre-syncope compared with asymptomatic tilt. In contrast, the respiratory related oscillatory component of MSNA variability HF_cMSNA_ increased during pre-syncope, when vagal activity was likely enhanced, highlighting the link between respiration and sympathetic efferent discharge activity [50] that can be mediated by vagal afferents [51].

Third, in our study the low frequency oscillation of systolic and diastolic blood pressure decreased during pre-syncope compared to asymptomatic tilt. Not surprisingly, the linear coupling between blood pressure and MSNA variability at 0.1 Hz was reduced, as quantified by K^2^_cMSNA-DAP_(LF). This points to a loss of synchronicity between the two oscillatory phenomena before syncope.

Fourth, in humans the physical properties of the vascular smooth muscle restrain the transduction of neurally mediated vasoconstriction to a narrow frequency range, with an optimal response at 0.1 Hz. Indeed, by using laser-Doppler flowmetry and sympathetic nerve electrical stimulation, Stauss and colleagues found that the largest decrease in skin blood flow was obtained during a stimulation frequency centered around 0.1 Hz [22]. In keeping with those observations are the results obtained from individuals with rare MSNA burst rate [23]. In these subjects, single sympathetic burst sequences occurred with a period of about 10 s. This further supports the concept that in humans there is a preferential frequency of sympathetic discharge activity to the vessels centered around 0.1 Hz [23].

### Limitations of the Study and Future Developments

A limitation of the present study is the small sample size, i.e., 10 individuals. Therefore, the *p* values we provide in the manuscript have to be interpreted within a simple exploratory purpose, aimed at setting the line for future ad-hoc studies possibly based on a greater population, rather than a way to draw definitive conclusions. 

It has to be highlighted that the current study was part of a complex and comprehensive protocol granted by the ESA, aimed at addressing the mid-term after-effects of −6° head-down bed confinement on several biochemical, morphological, cardiovascular and neural autonomic human variables. The overall protocol involved a number of European research groups, each addressing a specific sub-issue. ESA provided the facilities at DLR in Cologne and ESA doctors screened the volunteers’ health state and psychological profile. Their number was defined by ESA specialists. We had no say in the matter and could study only the 10 male subjects who were found suitable to enter the general protocol. 

On the other hand, it has to be pointed out that the uniqueness and completeness of the DLR facilities, including the presence of nurses and technical staff supporting the volunteers, the availability of advanced signals acquisition systems and the Tilt table test plus the lower body negative pressure devices, while enabling us to induce a safe pre-syncope episode in all the subjects, will make future protocols involving larger populations quite difficult without the help of these facilities. 

## 5. Conclusions

We suggest that the loss of synchronicity at 0.1 Hz of the sympathetic efferent nerve firing before pre-syncope might limit the capability of the vasculature to constrict in response to sympathetic firing. The response is likely related to an exceedingly attenuated sympathetic baroreflex modulation. Indeed, we observed reduced coupling between the cMSNA-DAP in the LF band during pre-syncope. Such a loss of effectiveness of the sympathetic vasomotor control would promote a progressive blood pressure fall and the onset of pre-syncope signs and symptoms upon standing, in spite of a vigorous compensatory tachycardia. 

Our findings could have implications above and beyond vasovagal syncope. Sympathetic activity in addition to regulating blood pressure and heart rate heralds cardiovascular risk. However, so far very few studies have investigated the oscillatory component of sympathetic activity and how this signal contributes to cardiovascular control and organ damage. Our study provides an impetus to study this property in more detail. 

## Figures and Tables

**Table 1:** Hemodynamic parameters, respiratory activity and sympathetic post-ganglionic discharge activity (MSNA) while supine (REST), during the asymptomatic phase of tilt (T_1_) and the period of tilt just preceding syncope (T_2_). Values are expressed as mean ± standard deviation.

Parameters	REST	T_1_	T_2_
RR (msec)	847 ± 99	609 ± 84 *	444 ± 87 §
SAP (mmHg)	131 ± 12	133 ± 10	114 ± 14 #§
DAP (mmHg)	76 ± 7	88 ± 8 *	71 ± 9 #
Resp (breaths/min)	17 ± 6	16 ± 3	18 ± 4
MSNA (bursts/min)	17 ± 6	30 ± 5 *	34 ± 9 §
MSNA (bursts/100 beats)	25 ± 8	31 ± 6	25 ± 5

* *p* < 0.05 REST vs T_1_. # *p* < 0.05 T_1_ vs T_2_. § *p* < 0.05 REST vs T_2_. RR: RR interval; SAP: systolic arterial pressure; DAP: diastolic arterial pressure; Resp: respiratory activity; MSNA: muscle sympathetic nerve activity.

**Table 2:** Arterial Baroreflex control of heart rate (α index and cBRSseq) and baroreceptor modulation of MSNA (sBRS) while supine (REST), during the asymptomatic phase of tilt (T_1_) and the period of tilt just preceding syncope (T_2_). Values are expressed as mean ± standard deviation.

Parameters	REST	T_1_	T_2_
α _LF_ (msec/mmHg)	14.0 ± 6.5	5.7 ± 3.0 *	1.8 ± 1.9 §
cBRSseq	17.5 ± 5.4	6.8 ± 3.7 *	2.8 ± 1.5 §
sBRS (% burst/mmHg)	−6.0 ± 3.3	−4.4 ± 1.2	−0.4 ± 0.9 #§

* *p* < 0.05 REST vs T_1._ # *p* < 0.05 T_1_ vs T_2._ § *p* < 0.05 REST vs T_2._

**Table 3:** Spectral parameters of autonomic function while supine (REST), during the asymptomatic phase of tilt (T_1_) and the period of tilt just preceding syncope (T_2_). Values are expressed as mean ± standard deviation.

Parameters	REST	T_1_	T_2_
RR_var_ (msec^2^)	2228 ± 710	1711 ± 1388	142 ± 223 #§
LF_RR_ (msec^2^) (n.u.)	797 ± 665 62.5 ± 25.3	1316 ± 1189 88.5 ± 11.3 *	100 ± 209 #§ 87.7 ± 8.9 §
HF_RR_ (msec^2^) (n.u.)	328 ± 200 35.9 ± 25.4	107 ± 144 9.3 ± 9.7 *	7 ± 14 #§ 5.8 ± 2.0 §
LF/HF_RR_	3.7 ± 3.7	28.3 ± 35.9 *	17.7 ± 9.2 §
LF_SAP_ (mmHg^2^)	3.3 ± 2.4	32.6 ± 16.9 *	10.0 ± 8.5 #
LF_cMSNA_ (bursts^2^·sec^−2^/Hz)	0.034 ± 0.014	0.058 ± 0.014	0.029 ± 0.019 #
K^2^_cMSNA-DAP_ (LF)	0.75 ± 0.19	0.90 ± 0.10	0.69 ± 0.15 #
HF_cMSNA_ (bursts^2^·sec^−2^/Hz)	0.0025 ± 0.0007	0.0028 ± 0.0008	0.0044 ± 0.0018 §

* *p* < 0.05 REST vs T_1_. # *p* < 0.05 T_1_ vs T_2_. § *p* < 0.05 REST vs T_2_. RR_VAR_: RR interval variance; LF_RR_: low frequency oscillatory component of RR variability; n.u.: normalized units; HF high frequency oscillatory component of RR variability; LF/HF_RR_: ratio between the low and high frequency oscillatory components of RR variability; LF_SAP_: low frequency oscillatory component of SAP variability; LF_cMSNA_: low frequency oscillatory component of calibrated MSNA; K^2^_cMSNA-DAP_(LF) coherence between the low frequency oscillatory components of calibrated MSNA and DAP variability; HF_cMSNA_: high frequency component of calibrated MSNA variability.

## References

[1] Brignole M., Moya A., De Lange F.J., Deharo J.-C., Elliott P.M., Fanciulli A., Fedorowski A., Furlan R., Kenny R.A., Martín A. (2018). 2018 ESC Guidelines for the diagnosis and management of syncope. Eur. Heart J..

[2] Costantino G., Perego F., Dipaola F., Borella M., Galli A., Cantoni G., Dell’Orto S., Dassi S., Filardo N., Duca P.G. (2008). Short- and long-term prognosis of syncope, risk factors, and role of hospital admission: results from the STePS (Short-Term Prognosis of Syncope) study. J. Am. Coll. Cardiol..

[3] Mosqueda-Garcia R., Furlan R., Md J.T., Fernandez-Violante R. (2000). The Elusive Pathophysiology of Neurally Mediated Syncope. Circulation.

[4] Furlan R., Alboni P., Mosqueda-Garcia R., Alboni P., Furlan R. (2015). Pathophysiology of Vasovagal Syncope: Conclusive Remarks. Vasovagal Syncope.

[5] Mosqueda-Garcia R., Furlan R., Fernandez-Violante R., Desai T., Snell M., Járai Z., Ananthram V., Robertson R.M., Robertson D. (1997). Sympathetic and baroreceptor reflex function in neurally mediated syncope evoked by tilt. J. Clin. Investig..

[6] Diedrich A., Paranjape S.Y., Robertson D. (2007). Plasma and Blood Volume in Space. Am. J. Med. Sci..

[7] Robertson D., Diedrich A., Chapleau M.W. (2012). Editorial on arterial baroreflex issue. Auton. Neurosci..

[8] Furlan R., Porta A., Costa F., Tank J., Baker L., Schiavi R., Robertson D., Malliani A., Mosqueda-Garcia R. (2000). Oscillatory Patterns in Sympathetic Neural Discharge and Cardiovascular Variables During Orthostatic Stimulus. Circulation.

[9] Diedrich A., Porta A., Barbic F., Brychta R.J., Bonizzi P., Diedrich L., Cerutti S., Robertson D., Furlan R. (2009). Lateralization of expression of neural sympathetic activity to the vessels and effects of carotid baroreceptor stimulation. Am. J. Physiol. Heart Circ. Physiol..

[10] Kamiya A., Hayano J., Kawada T., Michikami D., Yamamoto K., Ariumi H., Shimizu S., Uemura K., Miyamoto T., Aiba T. (2005). Low-frequency oscillation of sympathetic nerve activity decreases during development of tilt-induced syncope preceding sympathetic withdrawal and bradycardia. Am. J. Physiol. Heart Circ. Physiol..

[11] Schwartz C.E., Lambert E., Medow M.S., Stewart J.M. (2013). Disruption of phase synchronization between blood pressure and muscle sympathetic nerve activity in postural vasovagal syncope. Am. J. Physiol. Heart Circ. Physiol..

[12] Mosqueda-Garcia R., Robertson R.M., Hollister A.S., Biaggioni I., Netterville J.L., Robertson D. (1993). The Diagnosis and Treatment of Baroreflex Failure. N. Engl. J. Med..

[13] Furlan R., Magatelli R., Palazzolo L., Rimoldi A., Colombo S., Porta A. (2001). Orthostatic intolerance: different abnormalities in the neural sympathetic response to a gravitational stimulus. Auton. Neurosci..

[14] Jordan J., Shannon J.R., Black B.K., Costa F., Ertl A.C., Furlan R., Biaggioni I., Robertson D. (1997). Malignant Vagotonia Due to Selective Baroreflex Failure. Hypertension.

[15] Heusser K., Tank J., Luft F.C., Jordan J. (2005). Baroreflex Failure. Hypertension.

[16] Morillo C.A., Eckberg D.L., Ellenbogen K.A., Beightol L.A., Hoag J.B., Tahvanainen K.U., Kuusela T.A., Diedrich A.M. (1997). Vagal and Sympathetic Mechanisms in Patients With Orthostatic Vasovagal Syncope. Circulation.

[17] Ichinose M., Saito M., Fujii N., Kondo N., Nishiyasu T. (2006). Modulation of the control of muscle sympathetic nerve activity during severe orthostatic stress. J. Physiol..

[18] Barbic F., Heusser K., Marchi A., Zamunér A.R., Gauger P., Tank J., Jordan J., Diedrich A., Robertson D., DiPaola F. (2015). Cardiovascular parameters and neural sympathetic discharge variability before orthostatic syncope: Role of sympathetic baroreflex control to the vessels. Physiol. Meas..

[19] Mosqueda-Garcia R., Alboni P., Furlan R. (2015). Pathophysiology of Vasovagal Syncope: Role of Baroreceptor Mechanisms. Vasovagal Syncope.

[20] Cooke W.H., Rickards C.A., Ryan K.L., Kuusela T.A., Convertino V.A. (2009). Muscle sympathetic nerve activity during intense lower body negative pressure to presyncope in humans. J. Physiol..

[21] Furlan R., Montano N., Porta A., Alboni P., Furlan R. (2015). Cardiovascular Rhythms in Vasovagal Syncope. Vasovagal Syncope.

[22] Stauss H.M., Anderson E.A., Haynes W.G., Kregel K.C. (1998). Frequency response characteristics of sympathetically mediated vasomotor waves in humans. Am. J. Physiol..

[23] Diedrich A., Crossman A.A., Beightol L.A., Tahvanainen K.U., Kuusela T.A., Ertl A.C., Eckberg D.L. (2013). Baroreflex physiology studied in healthy subjects with very infrequent muscle sympathetic bursts. J. Appl. Physiol. (1985).

[24] Buehlmeier J., Mulder E., Noppe A., Frings-Meuthen P., Angerer O., Rudwill F., Biolo G., Smith S.M., Blanc S., Heer M. (2014). A combination of whey protein and potassium bicarbonate supplements during head-down-tilt bed rest: Presentation of a multidisciplinary randomized controlled trial (MEP study). Acta Astronaut..

[25] Mosqueda-Garcia R. (1996). Microneurography in neurological research. Am. Acad. Neurol..

[26] Pagani M., Lombardi F., Guzzetti S., Rimoldi O., Furlan R., Pizzinelli P., Sandrone G., Malfatto G., Dell’Orto S., Piccaluga E. (1986). Power spectral analysis of heart rate and arterial pressure variabilities as a marker of sympatho-vagal interaction in man and conscious dog. Circ. Res..

[27] Malik M. (1996). Heart rate variability: standards of measurement, physiological interpretation and clinical use. Task Force of the European Society of Cardiology and the North American Society of Pacing and Electrophysiology. Circulation.

[28] Parati G., Saul J.P., Di Rienzo M., Mancia G. (1995). Spectral Analysis of Blood Pressure and Heart Rate Variability in Evaluating Cardiovascular Regulation. A critical appraisal. Hypertension.

[29] Pomeranz B., Macaulay R.J., Caudill M.A., Kutz I., Adam D., Gordon D., Kilborn K.M., Barger A.C., Shannon D.C., Cohen R.J. (1985). Assessment of autonomic function in humans by heart rate spectral analysis. Am. J. Physiol..

[30] Barbic F., Perego F., Canesi M., Gianni M., Biagiotti S., Costantino G., Pezzoli G., Porta A., Malliani A., Furlan R. (2007). Early Abnormalities of Vascular and Cardiac Autonomic Control in Parkinson’s Disease Without Orthostatic Hypotension. Hypertension.

[31] Hamner J.W., Taylor J.A. (2001). Automated quantification of sympathetic beat-by-beat activity, independent of signal quality. J. Appl. Physiol. (1985).

[32] Wallin B.G., Burke D., Gandevia S. (1994). Coupling between variations in strength and baroreflex latency of sympathetic discharges in human muscle nerves. J. Physiol..

[33] Marchi A., Bari V., De Maria B., Esler M.D., Lambert E.A., Baumert M., Porta A. (2016). Calibrated variability of muscle sympathetic nerve activity during graded head-up tilt in humans and its link with noradrenaline data and cardiovascular rhythms. Am. J. Physiol. Regul. Integr. Comp. Physiol..

[34] Pagani M., Montano N., Porta A., Malliani A., Abboud F.M., Birkett C., Somers V.K. (1997). Relationship Between Spectral Components of Cardiovascular Variabilities and Direct Measures of Muscle Sympathetic Nerve Activity in Humans. Circulation.

[35] Porta A., Bari V., Bassani T., Marchi A., Pistuddi V., Ranucci M. (2013). Model-based causal closed-loop approach to the estimate of baroreflex sensitivity during propofol anesthesia in patients undergoing coronary artery bypass graft. J. Appl. Physiol. (1985).

[36] Magagnin V., Bassani T., Bari V., Turiel M., Maestri R., Pinna G.D., Porta A. (2011). Non-stationarities significantly distort short-term spectral, symbolic and entropy heart rate variability indices. Physiol. Meas..

[37] Bertinieri G., Di Rienzo M., Cavallazzi A., Ferrari A.U., Pedotti A., Mancia G. (1988). Evaluation of baroreceptor reflex by blood pressure monitoring in unanesthetized cats. Am. J. Physiol..

[38] Parati G., Di Rienzo M., Bertinieri G., Pomidossi G., Casadei R., Groppelli A., Pedotti A., Zanchetti A., Mancia G. (1988). Evaluation of the baroreceptor-heart rate reflex by 24-hour intra-arterial blood pressure monitoring in humans. Hypertension.

[39] Hart E.C., Joyner M.J., Wallin B.G., Karlsson T., Curry T.B., Charkoudian N. (2010). Baroreflex control of muscle sympathetic nerve activity: A nonpharmacological measure of baroreflex sensitivity. Am. J. Physiol. Heart Circ. Physiol..

[40] Marchi A., Bari V., De Maria B., Esler M., Lambert E., Baumert M., Porta A. (2016). Simultaneous Characterization of Sympathetic and Cardiac Arms of the Baroreflex through Sequence Techniques during Incremental Head-Up Tilt. Front. Physiol..

[41] Ei-Bedawi K.M., Hainsworth R. (1994). Combined head-up tilt and lower body suction: A test of orthostatic tolerance. Clin. Auton. Res..

[42] Sutton R., Brignole M. (2014). Twenty-eight years of research permit reinterpretation of tilt-testing: hypotensive susceptibility rather than diagnosis. Eur. Hear. J..

[43] Piccirillo G., Naso C., Moisè A., Lionetti M., Nocco M., Di Carlo S., De Laurentis T., Magrí D., Cacciafesta M., Marigliano V. (2004). Heart rate and blood pressure variability in subjects with vasovagal syncope. Clin. Sci..

[44] Ogoh S., Volianitis S., Raven P.B., Secher N.H. (2004). Carotid baroreflex function ceases during vasovagal syncope. Clin. Auton. Res..

[45] Jardine D.L., Melton I.C., Crozier I.G., English S., Bennett S.I., Frampton C.M., Ikram H. (2002). Decrease in cardiac output and muscle sympathetic activity during vasovagal syncope. Am. J. Physiol. Heart Circ. Physiol..

[46] Jardine D.L. (2013). Vasovagal syncope: new physiologic insights. Cardiol. Clin..

[47] Vaddadi G., Esler M.D., Dawood T., Lambert E. (2010). Persistence of muscle sympathetic nerve activity during vasovagal syncope. Eur. Hear. J..

[48] Fu Q., Verheyden B., Wieling W., Levine B.D. (2012). Cardiac output and sympathetic vasoconstrictor responses during upright tilt to presyncope in healthy humans. J. Physiol..

[49] Iwase S., Mano T., Kamiya A., Niimi Y., Fu Q., Suzumura A. (2002). Syncopal attack alters the burst properties of muscle sympathetic nerve activity in humans. Auton. Neurosci..

[50] Eckberg D.L., Nerhed C., Wallin B.G. (1985). Respiratory modulation of muscle sympathetic and vagal cardiac outflow in man. J. Physiol..

[51] Malliani A., Schwartz P., Zanchetti A. (1969). A sympathetic reflex elicited by experimental coronary occlusion. Am. J. Physiol..

